# Iliofemoral DVT thrombectomy: a dual-access approach to optimise inflow

**DOI:** 10.1186/s42155-025-00543-0

**Published:** 2025-05-15

**Authors:** Robert Wise, Zahi Qamhawi, Emma Wilton, Andrew Wigham

**Affiliations:** Department of Vascular and Interventional Radiology, John Radcliffe Hospital, Oxford, OX3 9DU UK

**Keywords:** Deep vein thrombosis, Aspiration thrombectomy, Iliofemoral, Inflow

## Background

One of the challenges of treating extensive iliofemoral deep venous thrombosis (DVT) with large-bore aspiration or mechanical thrombectomy is clearing the femoropopliteal and profunda femoral segments to attain good inflow. Most commonly, the popliteal vein (PV) is accessed, enabling antegrade clearance of the iliac and femoral segments. Antegrade profunda femoral vein (PFV) clearance may be possible via a popliteal-profunda communicator but can be challenging [1]. Jugular approach thrombectomy necessitates thrombus removal toward the cardiopulmonary vasculature, and device length may limit this approach.

We present a novel technique for iliofemoral DVT thrombectomy in cases with extensive inflow thrombosis using dual access of the ipsilateral great saphenous vein (GSV) and contralateral common femoral vein (CFV). GSV access allows antegrade thrombectomy of the iliac vein, while CFV access enables up-and-over retrograde thrombectomy of the inflow vessels comprising the femoral vein (FV), PV, and PFV. This technique is exemplified in two patients with lower limb DVT treated with the Lightning Flash 16 French aspiration system (Penumbra Inc, USA).

## Case 1

**Fig. 1:**
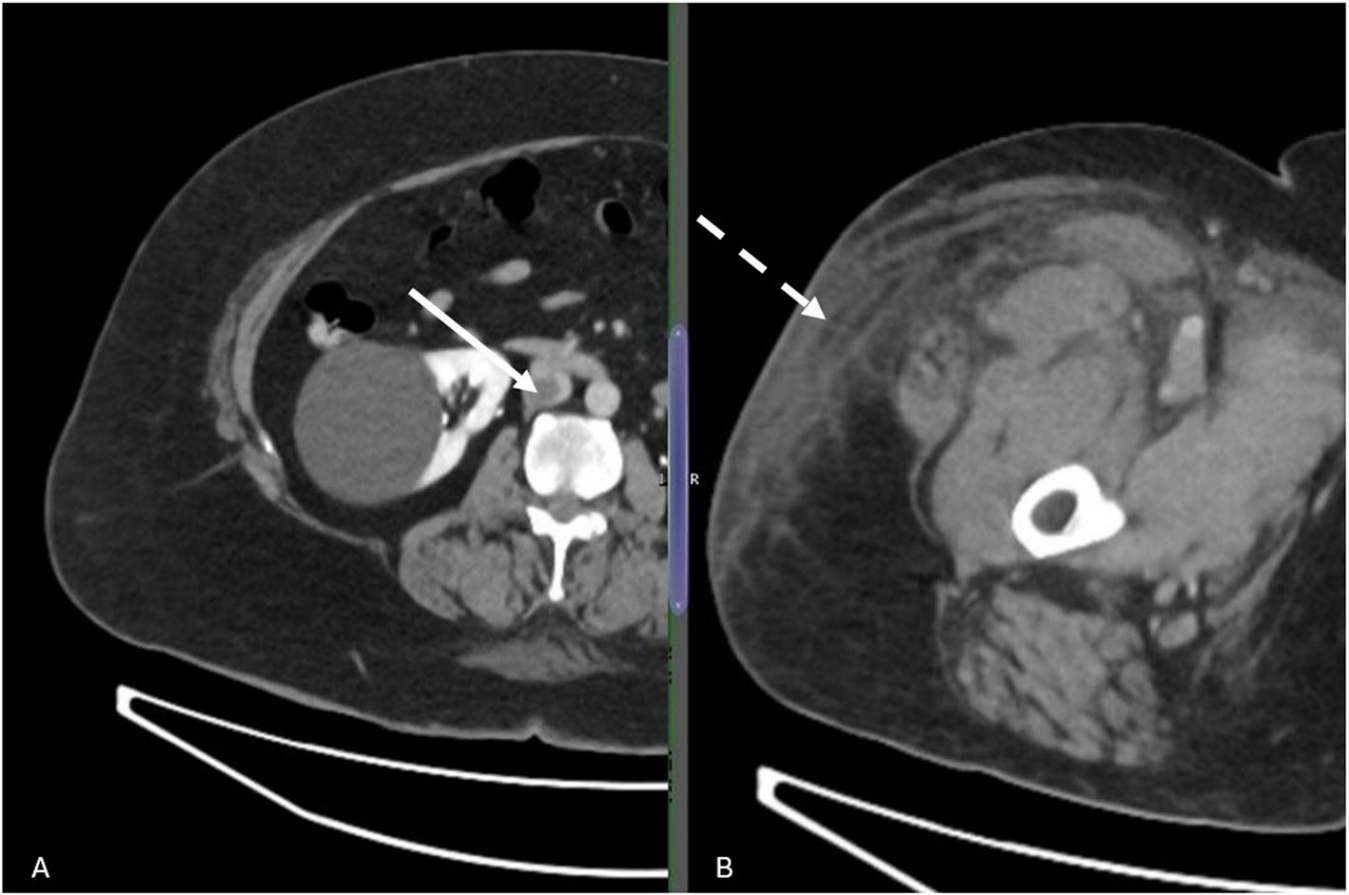
Computed Tomography imaging showing proximal extent of thrombus in the IVC (white arrow) **a**, and significant right lower limb superficial oedema (dashed arrow) **b**

A 69-year-old female presented with a two-day history of right lower limb swelling. Imaging (Fig. 1) revealed extensive DVT extending from the inferior vena cava (IVC) and right iliofemoral vein into the PFV, FV, and PV.

Aspiration thrombectomy was performed in an angiographic suite with the patient supine under general anaesthesia. Intravenous low-molecular-weight heparin was administered to maintain an activated clotting time > 200 s.

Ultrasound-guided access of the ipsilateral GSV and contralateral CFV was performed with insertion of 16 French DrySeal sheaths (Gore, USA). Venography and intravascular ultrasound (IVUS) confirmed thrombus extent. Antegrade thrombectomy of the IVC and right iliac vein was performed via the GSV using Lightning Flash, with temporary IVC embolic protection from contralateral access. Subsequently, the contralateral CFV sheath was advanced up-and-over into the right iliac vein while the ipsilateral sheath was retracted into the GSV. Retrograde thrombectomy of the right CFV, FV, PFV, and PV was then performed.

**Fig. 2:**
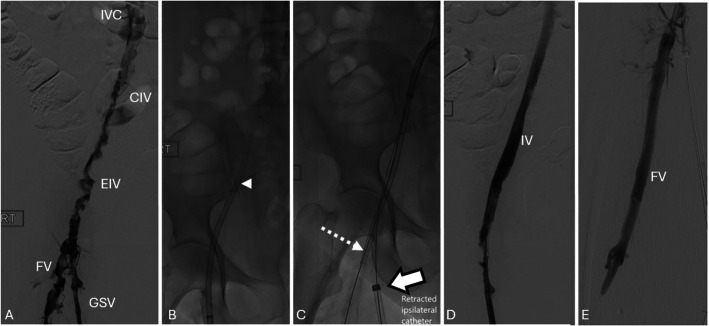
Dual-access technique for right lower limb thrombectomy using the right great saphenous vein (GSV) and contralateral left common femoral vein (CFV). **A** Venography from right GSV demonstrating thrombus in the inferior vena cava (IVC), right common iliac vein (CIV), external iliac vein (EIV), and femoral vein (FV). **B** Antegrade thromboaspiration of iliac vein and IVC using Lightning Flash (*arrowhead*). **C** Up-and-over contralateral sheath with retrograde downstream thrombectomy of inflow vessels using Lightening Flash (*dashed arrow*) with ipsilateral sheath retracted into GSV (s*olid arrow*). **D** Final venography demonstrating patency of IVC and right iliac vein. **E** Patent right femoral vein

Following thrombectomy, a right common iliac vein stenosis was treated with a 14 mm Atlas Gold high-pressure balloon (Becton Dickinson, USA). Final venography confirmed patency of the IVC, right iliac vein, PFV, FV, and PV (Fig. 2). The procedure took 70 min. The patient recovered uneventfully and was discharged on anticoagulation. Duplex ultrasound 48 h post-procedure confirmed continued patency of the IVC, right iliofemoral vein, and treated inflow veins.

## Case 2

**Fig. 3:**
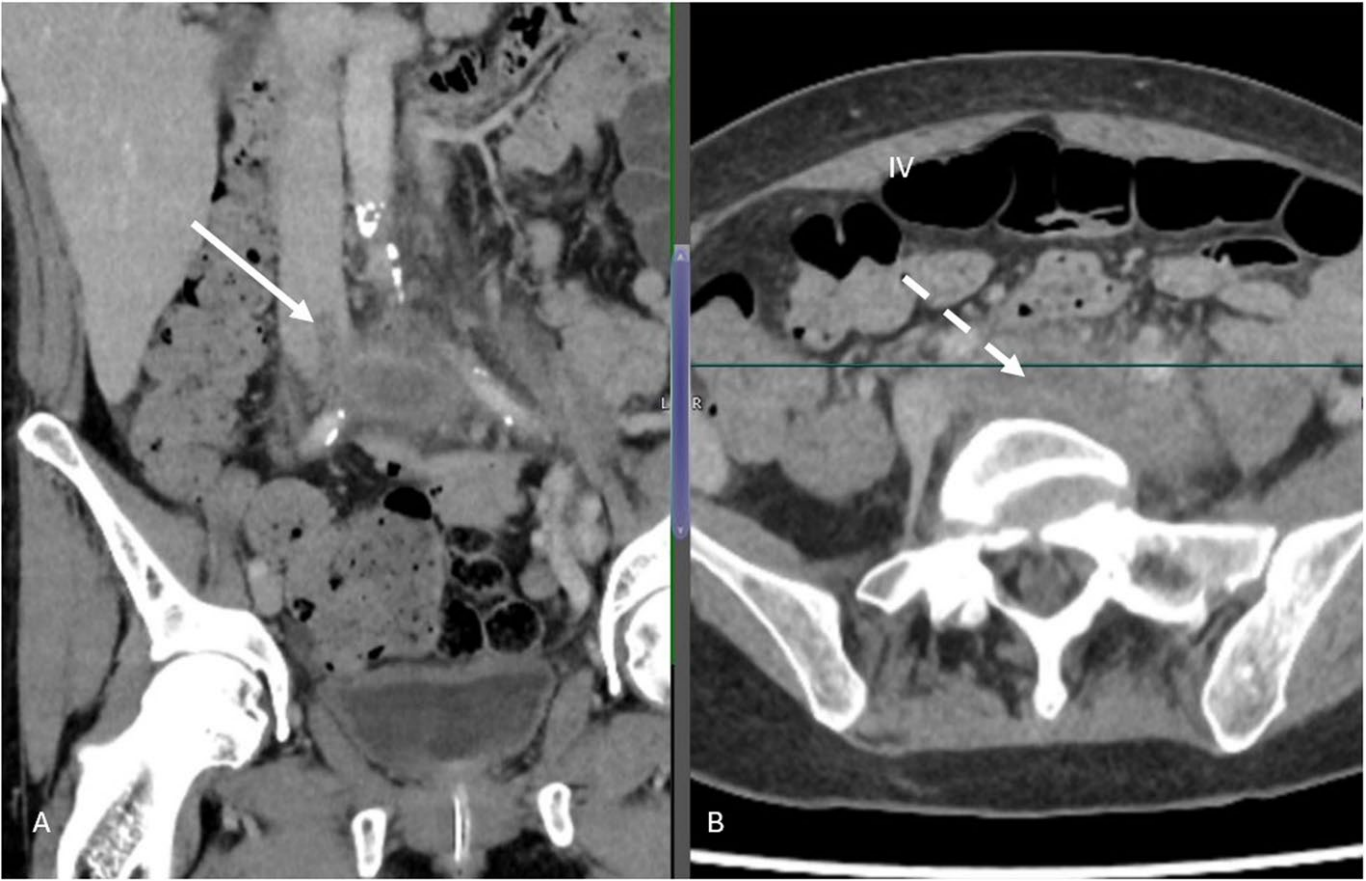
Computed Tomography imaging showing proximal extent of thrombus in the IVC (white arrow) **a**, and significant phlegmasia of the expanded left iliac vein (dashed arrow) **b**

A 56-year-old female presented with a three-week history of left leg swelling. Imaging (Fig. 3) revealed IVC and left iliofemoral vein thrombosis, with thrombus involving the PFV, FV, PV, and proximal tibial veins.

**Fig. 4:**
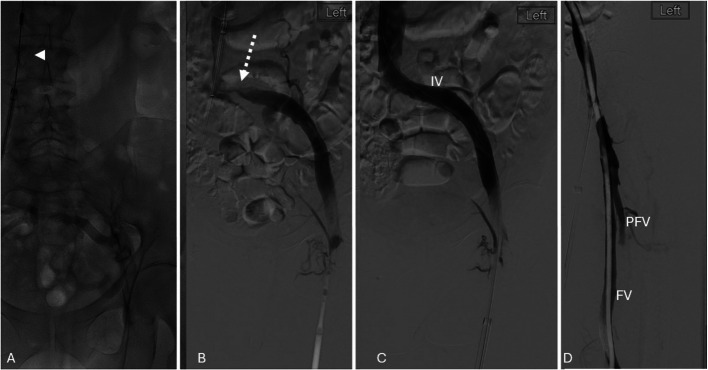
Dual-access technique for left lower limb thrombectomy using the left great saphenous vein (GSV) and contralateral right common femoral vein (CFV). **A** Venography demonstrating inferior vena cava (IVC), left common iliac vein (CIV) and external iliac vein (EIV) thrombosis. IVC and proximal CIV not opacified due to occlusion. Temporary embolic protection from contralateral access (*arrowhead*). **B** Iliocaval antegrade thrombectomy using Lightning Flash from GSV access revealing a May- Thurner compression point (dashed arrow) **C** Stent deployed from GSV access. **D** Up-and-over retrograde inflow thrombectomy of left profunda femoral vein (PFV), femoral vein (FV) and popliteal vein (not shown) using Lightning Flash (dashed arrow) from contralateral CFV access with GSV sheath retracted. Final venography demonstrating patency of inflow veins

The procedure mirrored Case 1, employing dual access of the ipsilateral GSV and contralateral CFV. Thrombectomy of the IVC and left iliac vein was performed with Lightning Flash, with contralateral temporary IVC embolic protection. Up-and-over retrograde thrombectomy addressed thrombus in the left PFV, FV, and PV via CFV access. A May-Thurner stenosis was treated with a 14 × 150 mm Abre stent (Medtronic, USA) deployed from the ipsilateral GSV access. Final imaging confirmed patency of the left iliofemoral stent and treated inflow veins (Fig. 4). The 90-min procedure resulted in an uneventful discharge on anticoagulation, with maintained stent and inflow vein patency at two-week follow-up.

## Conclusion

Optimising deep venous inflow during thrombectomy is increasingly recognised as essential, with poor inflow being a major predictor of venous re-occlusion or stent failure [2, 3]. This dual-access technique confers several advantages. First, it avoids accessing a major inflow vessel in the affected limb, reducing the risk of access-related complications or semi-occlusive large bore sheath placement. Instead, antegrade iliofemoral thrombectomy via accessing a superficial vein, such as the GSV, is feasible and less consequential in case of complications. Up-and-over thrombectomy then addresses inflow thrombosis down to the popliteal and potentially the tibial veins. The ability to retract sheath out of the CFV enables complete clearance of CFV from contralateral access. We prefer to avoid aspiration thrombectomy form a jugular access if possible due to device length, and pulmonary embolism risk if a ‘lollipop technique’ is required. Additionally, this technique allows supine thrombectomy, which offers anaesthetic advantages over prone approaches [4] A limitation of this technique is that not all thrombectomy devices can be deployed from a contralateral approach. Newer generations of aspiration devices such as Lightning Flash may address this.

## Data Availability

Not applicable.
